# Saliva – a pivotal player in the pathogenesis of oropharyngeal cancer

**DOI:** 10.1038/sj.bjc.6601869

**Published:** 2004-05-25

**Authors:** A Z Reznick, O Hershkovich, R M Nagler

**Affiliations:** 1 Department of Oral and Maxillofacial Surgery, Oral Biochemistry Laboratory and Salivary Clinic, Rambam Medical Center and Faculty of Medicine, Technion-Israel Institute of Technology, Haifa, Israel; 2 Department of Anatomy and Cell Biology, Faculty of Medicine, Technion-Israel Institute of Technology, Haifa, Israel

**Keywords:** cancer, oral, saliva, cigarette smoke, free radicals, redox metals, aldehydes

## Abstract

Oropharyngeal (OP) cancer, which is usually squamous cell carcinoma, is the most common head and neck malignancy and accounts for 2–4% of all new cancers. It is primarily induced by exposure to tobacco. The paradigm of cigarette smoke (CS)-induced OP cancer's pathogenesis is based on the assumption that a constant direct attack of various CS carcinogens causes widespread accumulating cellular and DNA aberrations in the OP mucosal cells, in turn eventually resulting in malignant transformation. However, there is never a direct contact between CS and the OP mucosa. Saliva, bathing the mucosa from the oral cavity to the larynx, always intervenes, and CS must first interact with saliva before it reaches the mucosa. The current study investigated the role of saliva in the pathogenesis of OP cancer. A synergistic effect of CS and saliva on oral cancer cells was demonstrated. This synergism is based on the reaction between redox active metals in saliva and low reactive free radicals in CS, which results in the production of highly active hydroxyl free radicals. Thus, when exposed to CS, salivary behavior is reversed and the saliva loses its antioxidant capacity and becomes a potent prooxidant milieu. The devastating role of CS-borne aldehydes was demonstrated as well. Based on these results and on our recent reports demonstrating that CS destroys various salivary components, including protective ones such as peroxidase, the most important salivary antioxidant enzyme, a comprehensive view of the pivotal role of saliva in the pathogenesis of CS-induced OP cancer is suggested.

Oropharyngeal (OP) cancer, which is usually squamous cell carcinoma, is the most common head and neck malignancy, having a worldwide incidence of over 300 000 new cases each year and accounting for 2–4% of all new cancers (Bross and Coombes, 1976; McGinnis and Foege, 1993; Collins *et al*, 1994; Ko *et al*, 1995; Piyathilake *et al*, 1995; Zheng *et al*, 1997; Nagler *et al*, 1999; Lippman and Hong, 2001). The commonly accepted paradigm for cigarette smoke (CS)-induced OP cancer pathogenesis is based on the assumption that a constant and direct attack of various CS carcinogens causes widespread accumulating cellular and DNA aberrations in the OP mucosa eventually leading to malignant transformation and cancer development. The CS-borne carcinogens are often thought to be free radicals. The process is initially expressed by dysplastic mucosal lesions which are then transformed into *in situ* carcinoma lesions, eventually resulting in full-blown infiltrating and metastasising OP cancer (Holleb *et al*, 1991). Chen *et al* (2002) demonstrated that chewing tobacco (areca quid) resulted in the formation of ROS in the oral cavity causing oxidative DNA damage to the surrounding tissues. Further support can be found in a report demonstrating that premalignant mucosal lesions and low-grade carcinomas express DNA aberrations following a pathway of exposure to free radicals (Sudbø *et al*, 2001). Moreover, Bloching *et al* (2001) recently found increased genotoxic activity in the saliva of smokers with a highly significant additional increase of genotoxicity measured in smoking and drinking individuals.

These and other recently published studies raise a significant question regarding the above OP cancer paradigm: Does saliva has a role in the pathogenesis of OP cancer and is there really a direct contact between CS carcinogens and the epithelium? The answer to the latter is ‘no.’ There is almost never a direct contact between CS and the mucosa because saliva is always present and continually bathes the mucosa from the oral cavity to the larynx, where CS is inhaled into the trachea and saliva is swallowed into the oesophagus.

We explored the effect of CS in the presence of saliva on oral cancer cells (SCC-25), as this cell line is one the most often used cellular model of OP cancer. Our results and their implications for the understanding of CS-induced OP cancer are presented and discussed, and an overall schematic algorithm for the role of saliva in the pathogenesis of this cancer is suggested.

## MATERIALS AND METHODS

### Study design

Oral cancer cells (SCC-25) were exposed to CS and its effect on cell survival and carbonyls production (demonstrating oxidation induced structural aberrations) was analysed. The cells were exposed for a period of up to 90 min during which analysis was performed at various time points. The cells were incubated while exposed to CS in either PBS alone or in a mixture of PBS and saliva, where the PBS was supplemented with 30% (v v^−1^) whole saliva.

The study design was divided into three parts: first, an observed lethal synergistic effect of saliva and CS on the exposed cells was explored with respect to extent and kinetics. Second, a similar analysis was performed with two additional different types of saliva: one secreted specifically from the parotid gland and the other from the submandibular/sublingual (Sm/Sl) glands. Third, various agents were added to the saliva and the effect of CS on the cells was analysed to explore the nature of possible participating injurious components in saliva and/or in smoke which might be responsible for the effect observed. The additions included Glutathione (GSH) 10 mM and/or Desferal (DES) 10 mM, *N*-Acetyl-L-cystein (NAC) 10 mM, Ascorbic acid (Asc) 10 mM, hydroxocobalamin (OH-CO) 10 mM or Ebselen 1 mM, all of which were purchased from the Sigma Chemical Co St Louis, MO, USA. All experiments were repeated for three to five times and the mean values of the reproducible results are presented.

### Saliva collection

Saliva (whole saliva, parotid saliva and SM/SL saliva) was collected under both resting and stimulated conditions from six healthy volunteers (three males and three females, aged 21–47 years) in a quiet environment between 0800 and noon as previously described (Nagler *et al*, 1996, 2000, 2001, 2002a). No oral stimulus was permitted for 90 min prior to collection. A Carlson Crittenden cup was used to obtain parotid saliva. Submandibular/sublingual saliva was collected by standardized gentle suction from the floor of the mouth. Following collection under resting conditions a stimulated collection of saliva was performed, for which a 2% citric acid solution was applied to the tongue dorsum bilaterally at 30 s intervals. Following collection, the saliva was immediately centrifuged at 800 **g** at 4°C for 10 min to remove squamous cells and cell debris, and was then used in the experiments.

### Exposure of the SCC-25 cells in medium with/without saliva to CS

The cigarettes used in this study were commercial cigarettes containing 14 mg of tar and 0.9 mg of nicotine (Time Cigarettes, Dubek Ltd, Tel Aviv, Israel). To expose the saliva to CS a cigarette from which the filter tip had been removed was attached to a Cambridge filter (capable of removing particles >0.1 mm in diameter), which was combined with a vacuum system to enable the inhalation of gas-phase CS inside sealed 250 ml flasks containing the SCC-25 cells in 12–15 ml PBS with/without saliva as previously described (Nagler *et al*, 2001). A reproducible vacuum was created in the flask and, upon opening the vacuum to the lighted cigarette for 5 s, 80–100 ml of CS ‘puffs’ were drawn into the flask. After half the cigarette had been ‘inhaled,’ the flasks were incubated for 20 min at 37°C in a metabolic shaker, and then taken for further inhalation.

### Cells, culture conditions and cellular survival

SCC-25 (human oral squamous cell carcinoma cells from the American Type Culture Collection, Rockville, MD, USA) were grown in 85% DMEM-Ham's F-12 media. Cultures also contained 0.4 *μ*g ml^−1^ hydrocortisone, 15% heat-inactivated FBS, 100 U ml^−1^ penicillin, 10 *μ*g ml^−1^ streptomycin, and 0.25 *μ*g ml^−1^ amphotericin B and were grown at 37°C in 95% air and 5% CO_2_. The medium was changed every 3 to 5 days. Routine subculture was accomplished using 0.25% trypsin and 0.1% EDTA. Cells were collected by using 0.25% trypsin and 0.1% EDTA, then resuspended in phosphate-buffered saline (PBS: KCl 2.7 mM, KH_2_PO_4_ 1.5 mM, NaHPO_4_ 8 mM, and NaCl 136.9 mM, pH 7.0) and pelleted at 1500 rpm for 12 min in 1.5 ml microfuge tubes. The cells collected were used immediately for Western activity gel and for viability analysis where SCC-25 cells were plated into 100 mm dishes at a density of 1 × 10^6^ cells/plate in full media. The viability of SCC-25 cells was measured at various times by Trypan Blue exclusion test, both in exposed and control cells.

### Protein carbonyl assay

SCC-25 cells from exposed and control mediums were washed twice in PBS after centrifugation at 2000 rpm for 2 min to remove the saliva and components of the incubation medium. The cells were then centrifuged at 14000 rpm for 1 min and lysed in lysis buffer containing 20 mM tris buffer pH 7.4, 1 mM EGTA, 1 mM PMSF, 50 *μ*M NaVO_4_, 50 mM NaF, by sonication for 10 s. The solution was centrifuged at 14000 rpm for 1 min and the protein-containing liquid phase was used. Carbonyl analysis using SDS–PAGE and Western Blot anti-DNP antibodies was carried out with the OxyBlot Kit (Sigma, NY, USA), according to the manufacturer's instructions as previously described (Reznick *et al*, 1997; Nagler *et al*, 2001).

### Statistical evaluation

The results for the statistical evaluation were taken from the control subgroup (SCC-25 cells in PBS medium) and from the study subgroups (with/without saliva and/or exposure to CS and/or various additions of exogenous reagents including GSH, NAC, DES, Asc, OH-CO or Ebselen). Means, standard deviations and standard errors were computed. The results between the subgroups were analysed with one-way analysis-of-variance. The means between each pair of means was analysed with *T*-test for paired differences. The means between each two subgroups was analysed with two sample *T*-test for differences in means.

## RESULTS

### Exposure of SCC-25 cells to CS with/without whole saliva

**Figure 1:**
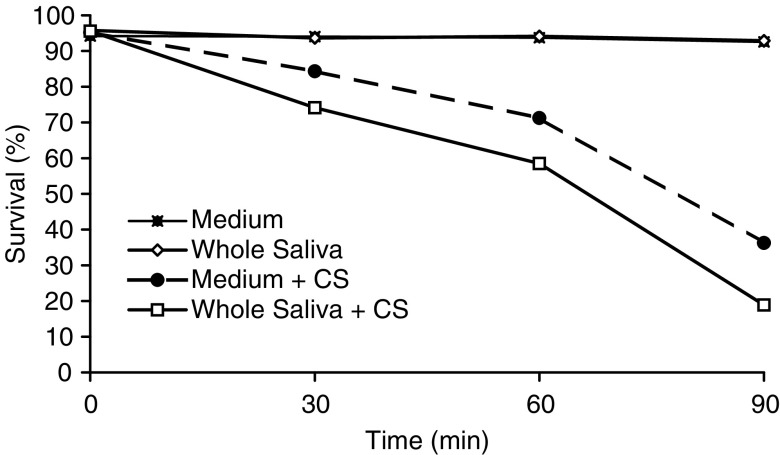
Survival rate of SCC-25 cells exposed to cigarette smoke (CS) and whole saliva collected under *resting conditions*. SCC-25 incubated at 37°C in PBS alone. SCC-25 incubated at 37°C in the presence of saliva. SCC-25 incubated at 37°C in the presence of PBS and exposed to CS. SCC-25 incubated at 37°C in the presence of saliva and exposed to CS.

**Figure 2:**
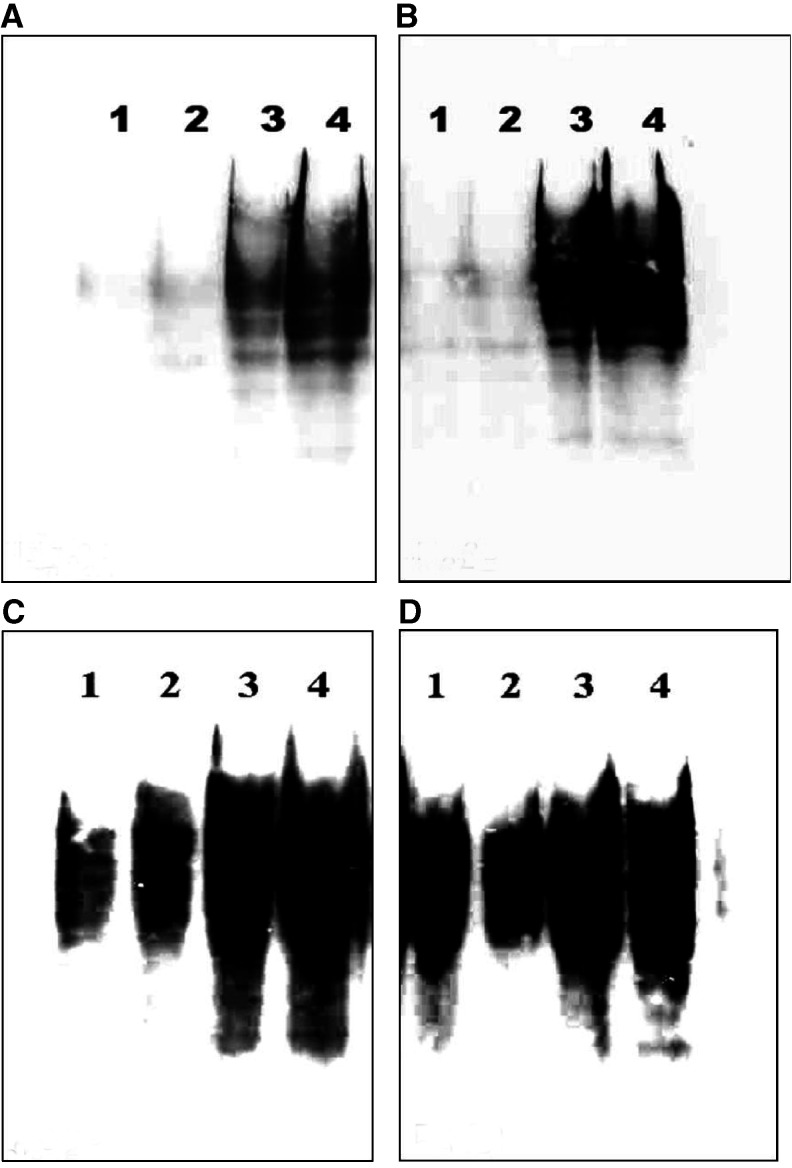
Salivary mediatory effect on cigarette smoke induced carbonylation pattern (oxidation level) of SCC-25 cells measured by Western blotting with anti-DNP antibody after 15 min (**A**), 30 min (**B**), 60 min (**C**) and 90 min (**D**). *Lane 1* shows the protein cabonylation pattern of SCC-25 cells incubated at 37°C in PBS alone. *Lane 2* shows the protein cabonylation pattern of SCC-25 cells incubated in the presence of saliva. *Lane 3* shows the protein cabonylation pattern of SCC-25 cells incubated at 37°C in PBS and exposed to CS. *Lane 4* shows the protein cabonylation pattern of SCC-25 cells incubated at 37°C in the presence of saliva and exposed to CS.

During a 90-min incubation period of SCC-25 cells in PBS alone or in PBS supplemented with 30% (v v^−1^) saliva, no survival loss was observed (Figure 1

). Protein oxidation as demonstrated by carbonylation was not observed at 15 min or at 30 min and only mildly at 60 min and later (Figure 2

). However, after 90 min of exposure to CS, a time-dependent reduced survival of the SCC-25 cells (in PBS) and an increase in the protein oxidation level as measured by Western Blot for carbonyls were observed (survival loss of 61.6% (*P*<0.01)). The addition of saliva to the PBS while exposed to CS resulted in a significant lethal synergistic effect as demonstrated by the 80.4% (*P*<0.01) survival loss and a profound protein oxidation (Figures 1 and 2). The salivary enhancement of the lethality induced by CS exposure to the cells was dose-dependent and statistically significant at all time points examined, from 30 to 90 min. It was also accompanied by a dose-dependent increase in protein oxidation level as measured by carbonylation at all time points examined – 15–90 min (Figure 2).

### Exposure of SCC-25 cells to CS with/without various types of saliva

Figure 3

**Figure 3:**
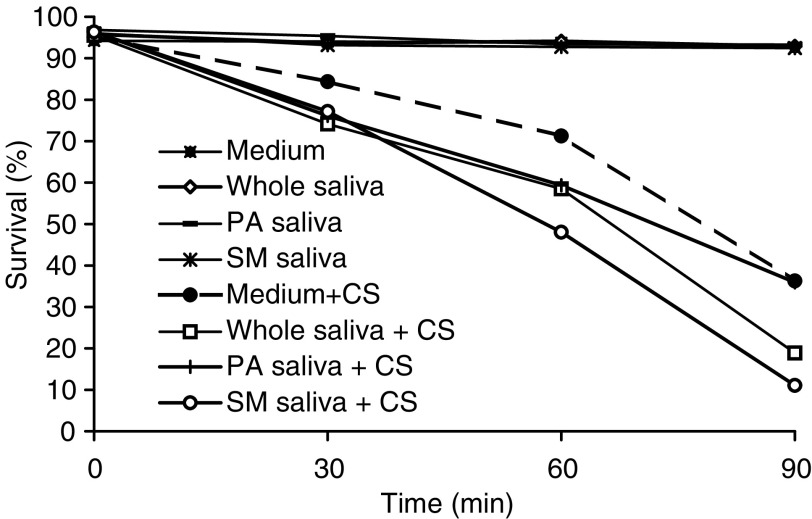
Survival rate of SCC-25 cells exposed to cigarette smoke (CS) and differentiated saliva collected under *resting conditions*. SCC-25 cells incubated at 37°C in PBS alone. SCC-25 cells incubated at 37°C in the presence of whole saliva. SCC-25 cells incubated at 37°C in the presence of parotid saliva. SCC-25 cells incubated at 37°C in the presence of Sm/Sl saliva. SCC-25 cells incubated at 37°C in PBS alone and exposed to CS. SCC-25 cells incubated at 37°C in the presence of whole saliva and exposed to CS. SCC-25 cells incubated at 37°C in the presence of parotid saliva and exposed to CS. SCC-25 cells incubated at 37°C in the presence of Sm/Sl saliva and exposed to CS.

shows the survival results obtained for SCC-25 cells exposed to CS with/without two other types of saliva – parotid and Sm/Sl saliva, collected under *resting conditions* in addition to the results obtained following the exposure of the cells to CS in the presence of whole saliva. As is clearly noted, both parotid and Sm/Sl saliva induce this lethal synergism phenomenon. Parotid saliva significantly enhanced the lethal effect of CS when added to the PBS (as compared to PBS alone) at 30 min but not at 60 or 90 min. The Sm/Sl was found to be the most cytotoxic saliva examined and its synergistic effect was significantly more lethal than that induced by parotid saliva or by whole saliva at both 60 and 90 min and especially more than the effect induced by PBS alone at 30, 60 and 90 min. Thus, at 90 min, while cell survival rate following exposure to CS in the presence of PBS alone was reduced by 61.6%, in the presence of PBS supplemented with whole saliva and PBS supplemented with Sm/Sl saliva, it dropped by 80.4 and 88.6% respectively (*P*<0.05) (Figure 3). A similar experiment was conducted with whole, parotid and Sm/Sl saliva secreted under *stimulated conditions* in which the saliva is naturally diluted (Figure 4

**Figure 4:**
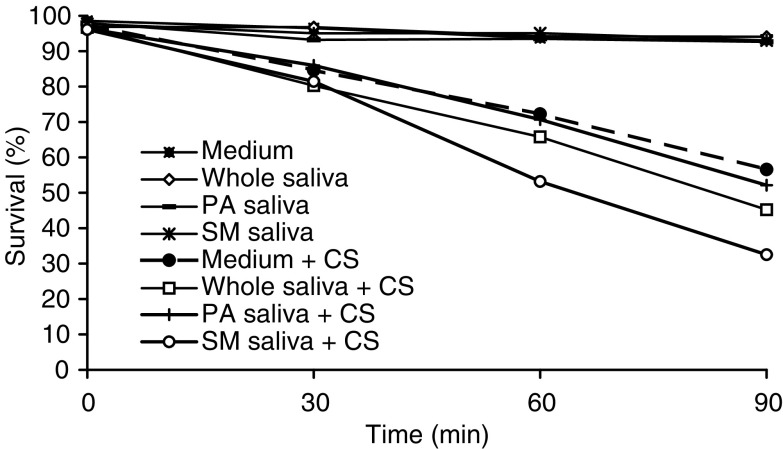
Survival rate of SCC-25 cells exposed to cigarette smoke (CS) and differentiated saliva collected under *stimulated conditions*. SCC-25 cells incubated at 37°C in PBS alone. SCC-25 cells incubated at 37°C in the presence of whole saliva. SCC-25 cells incubated at 37°C in the presence of parotid saliva. SCC-25 cells incubated at 37°C in the presence of Sm Sl saliva. SCC-25 cells incubated at 37°C in PBS alone and exposed to CS. SCC-25 cells incubated at 37°C in the presence of whole saliva and exposed to CS. SCC-25 cells incubated at 37°C in the presence of parotid saliva and exposed to CS. SCC-25 cells incubated at 37°C in the presence of Sm/Sl saliva and exposed to CS.

). This experiment demonstrated an almost identical – although somewhat more moderate – pattern of synergistic effects. Parotid saliva did not demonstrate a synergistic effect at any of the time points examined while that the Sm/Sl saliva was found to be most cytotoxic, also under stimulated conditions. Thus, at 90 min following exposure to CS in the presence of whole saliva and Sm/Sl saliva, the survival rate of the cells dropped by 53.3 and 66.2% respectively (*P*<0.05) (Figure 4). The results obtained for one of the subjects examined, a healthy 24-year old male, in whom this phenomenon was profoundly expressed under both resting and stimulated conditions, are presented in Figures 5 and 6

**Figure 5:**
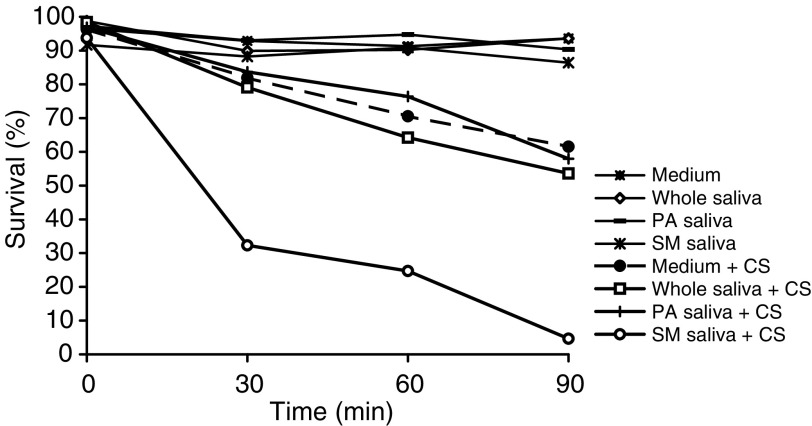
Survival rate of SCC-25 cells exposed to cigarette smoke (CS) and differentiated saliva (whole, parotid and Sm/Sl) collected under *resting conditions*. These cellular survival rates represent one case of a healthy 24 year old male, in whom the cytotoxic effect of saliva and more so of Sm/Sl saliva was profoundly expressed.

**Figure 6:**
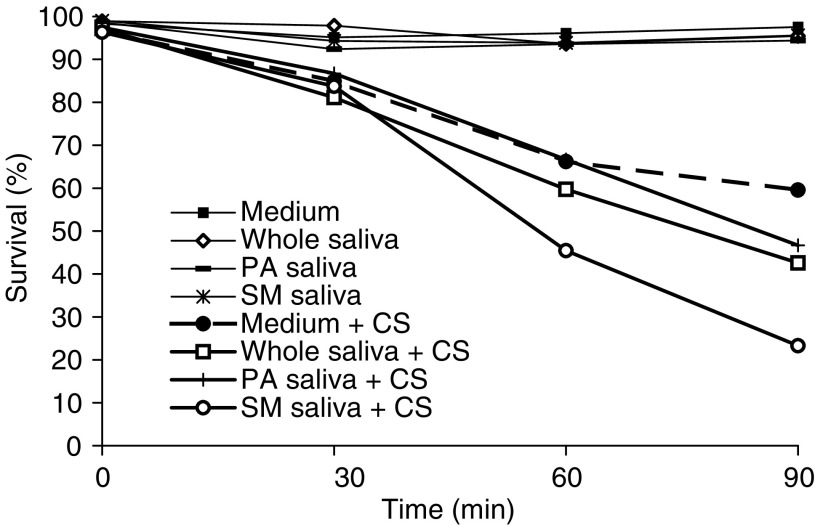
Survival rate of SCC-25 cells exposed to cigarette smoke (CS) and differentiated saliva (whole, parotid and Sm/Sl) collected under *stimulated conditions*. These cellular survival rates represent one case of a healthy 24 year old male, in whom the cytotoxic effect of saliva and more so of Sm/Sl saliva was profoundly expressed.

. Clearly, the Sm/Sl saliva is mostly cytotoxic and more so when secreted under resting conditions (only 5% survival) than when secreted under stimulated conditions (Figures 5 and 6).

### Protection against CS and saliva effect on SCC-25 cells with various agents

**Figure 7:**
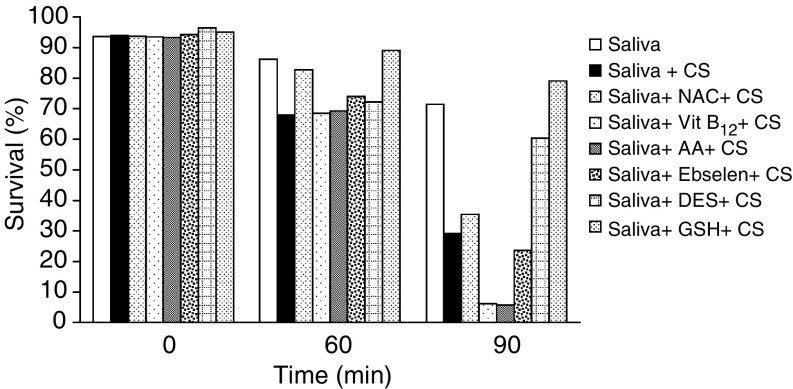
Modulatory effects of several antioxidants on survival rate of SCC-25 cells exposed to cigarette smoke (CS) in the presence of whole saliva collected under *resting conditions*. The survival rate values of SCC-25 cells exposed to CS, whole saliva and the added antioxidants: GSH 10 mM, NAC 10 mM, DES 10 mM, Asc (AA) 10 mM, OH-CO (Vit B_12_) 10 mM and Ebselen 1 mM, were measured at base line (0 time), 60 and 90 min as shown above.

To examine agents in the saliva and/or smoke that might be responsible for the synergism observed, various additions were made to the PBS containing whole saliva (secreted under resting conditions) prior to their exposure to CS for 90 min. These included GSH 10 mM, NAC 10 mM, Des 10 mM, Asc 10 mM, OH-CO 10 mM or Ebselen 1 mM (Figure 7

).

It was found that when saliva was added to the PBS, the cell survival rate at 60 min dropped by 27.7% (*P*<0.05) and at 90 min by 72.1.1% (*P*<0.01). This loss was prevented at 60 min by two agents known to prevent the injurious effects of aldehydes, the thiols GSH and NAC. Glutathione totally protected the cells at both 60 and 90 min, while NAC partially protected them at 60 but not at 90 min. At 90 min, however, the iron chelator DES demonstrated a protective capacity, while as noted, it did not at 60 min; at 90 min the 15.5% reduction in cell survival, which was observed in the DES (plus CS) group, was not statistically significant. Thus, the survival rates of these three groups (no CS, CS plus DES, CS plus GSH), while not being significantly different with respect to one other, were significantly higher (*P*<0.01) than those of the other five CS-exposed groups evaluated, that is, saliva added alone or supplemented with NAC, Asc, OH-CO (Vit B_12_) or Ebselen. The survival rates following the addition of OH-CO and Asc were reduced extensively by 91.4 and 92%, respectively (*P*<0.05) (Figure 7).

**Figure 8:**
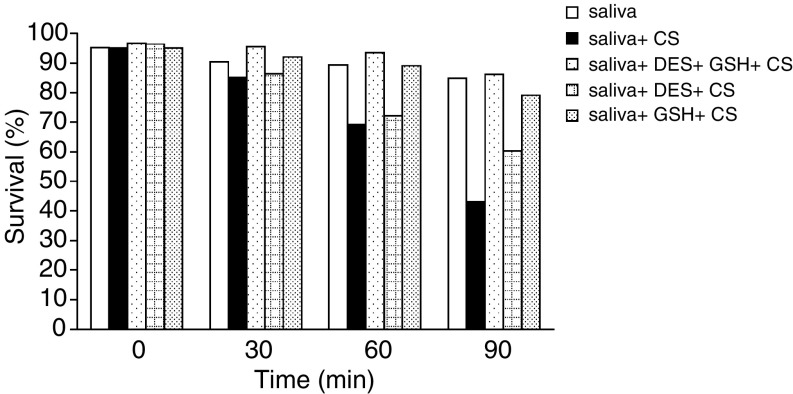
Modulatory effects of GSH 10 mM and/or DES 10 mM on survival rate of SCC-25 cells exposed to cigarette smoke (CS) in the presence of whole saliva collected under *resting conditions*. Measurements were performed at base line (0 time) and at 30, 60 and 90 min.

**Figure 9:**
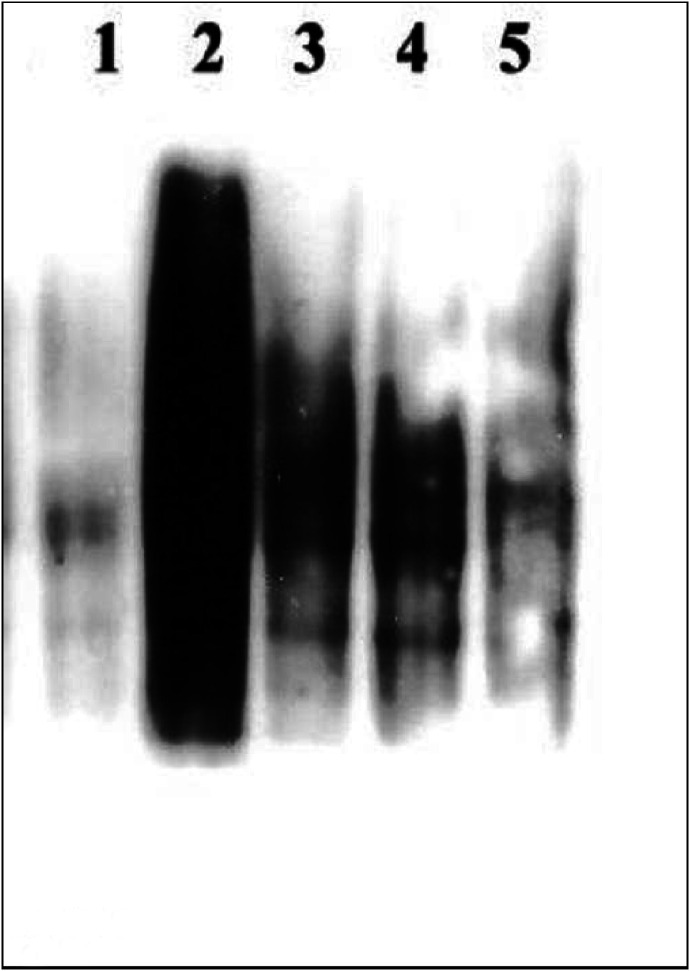
Modulatory effects of DES 10 mM and/or GSH 10 mM on the carbonylation pattern (oxidation level), which is induced by exposure of SCC-25 cells to cigarette smoke (CS) in the presence of whole saliva collected under *resting conditions*. This was performed on a Western blotting with anti-DNP antibody, at 15 min post-CS exposure. *Lane 1* shoes the protein cabonylation pattern of SCC-25 cells incubated at 37°C in the presence of saliva (with no CS exposure). *Lane 2* shoes the protein cabonylation pattern of SCC-25 cells incubated at 37°C in the presence of saliva and exposed to CS. *Lane 3* shoes the protein cabonylation pattern of SCC-25 cells incubated at 37°C in the presence of saliva+DES 10 mM and exposed to CS. *Lane 4* shoes the protein cabonylation pattern of SCC-25 cells incubated at 37°C in the presence of saliva+GSH 10 mM and exposed to CS. *Lane 5* shoes the protein cabonylation pattern of SCC-25 cells incubated at 37°C in the presence of saliva+DES 10 mM+GSH 10 mM and exposed to CS.

Thus, as it was observed that the most potent protective agents were GSH and DES, another study was conducted in which only they were examined for protective capacity, alone or in combination. The results are given in Figure 8

, where it can be seen that at 30, 60 and 90 min, the survival rates of cells exposed to CS in the presence of saliva alone (without additions of GSH or DES) dropped to 89.4% (NS), 72.6% (*P*<0.01) and 45.2% (*P*<0.01) respectively. It can also be seen that both GSH and DES efficiently protected the cells from the synergistic effect though GSH was somewhat more protective; however, the combination of both GSH and DES was the most efficient, resulting in absolutely no cell survival loss at any of the time points examined. Moreover, as previously demonstrated for the synergism itself, which paralleled the amount of carbonyl oxidation (Figures 1 and 2), the protection of SCC-25 cells against the lethal effect of CS in the presence of saliva was accompanied by a reduction in the carbonyl oxidation level. Thus, the level of oxidation prevention correlated with the level of cell death prevention and was absolute when no cell survival lost occurred following the addition of both GSH and DES, as previously demonstrated (Figure 9

).

## DISCUSSION

A lethal synergistic effect of CS and saliva on oral cancer cells was demonstrated for the first time. Its importance is due both to its novelty and to its possible biological and pathological significance. Salivary enhancement of CS effects might explain the high prevalence of CS-induced OP cancer. In addition, saliva is always considered an efficient protective medium – an antibacterial, antiviral, anticariogenic, antioxidative, mechanical and thermal protector, etc (Nagler *et al*, 2002b). However, the current study demonstrates that in the ‘wrong circumstances’ saliva becomes highly deleterious, such as when it is exposed to CS. The widely accepted paradigm regarding OP cancer is that it results from a CS-induced, continuous, step-wise increase of accumulative DNA aberrations which eventually result in transforming healthy cells into low malignant cancer cells. The ongoing injurious process further transforms those cells into highly aggressive invading cancer cells (Nagler *et al*, 1999; Nagler, 2002). Therefore, we chose SCC-25 cells for the current study as they are the most widely studied oral cancer cellular model. We wanted to determine whether exposing them to CS would induce further oxidative stress and injury, as is expected to occur in patients. Since it is thought that malignant cells are transformed into highly malignant cells following further injuries induced by carcinogens as free radicals, we also wanted to investigate whether saliva had a role in the process.

Figures 3, 4, 5 and 6 show that the Sm/Sl glands secrete very highly cytotoxic saliva as compared with that secreted by the parotid gland. An explanation may be that most of the balancing antioxidants in saliva, such as peroxidase, superoxide dismutase and uric acid, are secreted by the parotid and not the Sm/Sl glands, as was recently demonstrated (Nagler *et al*, 2002a). Accordingly, the injurious agents that produce the lethal oxidative stress, as shown both by the survival and carbonylation assays (Figures 1 and 2), cannot be neutralized in Sm/Sl saliva and are consequently very harmful. Our aim was to discover the injurious agents responsible. They were found to be innocent only if there was no CS present, as shown by the control group, in which the cells were incubated in the presence of saliva but without exposure to CS, with the result that there was no reduction in cellular survival or in carbonyl formation (Figures 1, 2, 3, 4, 5 and 6). Moreover, under stimulated conditions, the cellular loss inflicted was limited, since stimulated saliva is diluted, on the one hand, and its parotid-to-Sm/Sl ratio is increased, on the other (parotid secretion is increased by stimulation substantially more than Sm/Sl secretions) (Figures 4 and 6). Thus, we concluded that these injurious agents originate mainly in the Sm/Sl and not in the parotid saliva.

The fact that the Sm/Sl saliva is by far the most cytotoxic (Figures 5 and 6) is discouraging because Sm/Sl is by far the most important maintenance saliva secreted in large volumes at all times, while parotid saliva is substantially secreted mainly following stimulation, such as ingestion. Accordingly, the OP mucosa is bathed by Sm/Sl saliva most of the time and the injurious synergistic process may occur whenever a cigarette is smoked.

To examine the synergistic mechanism we added agents to the saliva prior to CS exposure. Neither Ebselen nor OH-CO forestalled any of the injurious effects of CS in the presence of saliva. Accordingly, we concluded that neither reactive nitrogen species (RNS), whose activity is inhibited by Ebselen, nor HCN, which is the CS agent inhibited by OH-CO, is involved. This experiment was based on previous reports that pointed to RNS and HCN as CS injurious agents. Reactive oxygen species have been intimately related to carcinogenesis and perhaps in the oral cavity as well (Ohashi *et al*, 1999; Lala and Chakraborty, 2001; Brennan, 2003).

Another line of research focused on the possible role of CS-borne aldehydes (such as acroline or acetaldehyde) which are known to mediate the CS-inflicted damage in other systems, such as on plasma proteins (Reznick *et al*, 1992). The addition of the biological thiol GSH (often recognised as the primordial antioxidant), intended to prevent aldehyde damage, substantially protected the cells in our system at all time points examined (Figures 7, 8 and 9). The fact that NAC, another thiol and a nontoxic GSH-prodrug, did not protect the cells as efficiently may be explained by the fact that it is readily accepted by the cells, while GSH is not, thus leaving GSH outside the cell to neutralize the CS-attacking aldehydes (Gmunder *et al*, 1991). Aldehydes are known CS components that do not exist in saliva. They never react with any known salivary components and as noted previously, they directly attack macromolecules in other systems.

In contrast, redox active metals such as iron, which are prevalent in saliva (Nagler *et al*, 1997; Nagler and Baum, 2003) in the presence of H_2_O_2_, and other low active ROS found in CS, such as suproxide dismutase radicals, participate in the Haber–Weiss and Fenton reactions in which deleterious highly reactive hydroxyl (·OH) free radicals are produced. Accordingly we postulated that these metals may be responsible for the synergistic mechanism. In order to examine this assumption, we added the potent iron chelator DES to the saliva prior to exposing the cells to CS. Indeed, the addition of DES substantially prevented damage nearly as efficiently as GSH, although only at 90 min. A possible explanation may be that an earlier aldehyde attack paves the way for a later one inflicted by salivary iron-related ROS, a kind of ‘cross-talk’ between the direct aldehyde-related and synergistic metal-related pathways of attack. Furthermore, the fact that the addition of Asc resulted in enhanced cellular death is also in accord with the suggested mechanism, as it is known that redox active iron in the presence of Asc participates in Fenton and Haber–Weiss reactions to transform low reactive free radicals into highly reactive hydroxyl free radicals (Halliwell and Foyer, 1976). Moreover, neither Asc nor DES modulated the effect of CS when the cells were not incubated in the presence of saliva but only in PBS (data not shown). Further significant credence for the mechanism suggested is found in the reported existence of redox metals (iron and copper) in saliva and of their deleterious role in the pathogenesis of the damage inflicted on salivary glands by ionising irradiation (another source of low reactive free radicals) (Nagler *et al*, 1997; Nagler and Baum, 2003). Finally, major support for the suggested mechanism can be found in a recent report which demonstrated that in humans the Sm/Sl saliva contains much higher concentrations of iron than does the parotid saliva, especially when secreted under resting conditions (Nagler *et al*, 2002b). This undoubtedly adds to explanation given previously for the profound cytotoxic behavior of Sm/Sl saliva as compared with parotid saliva, as well as for the general role of salivary redox metals, such as iron, in the underlying mechanism of the synergism phenomenon.

At this point we concluded that the two major underlying components of the mechanism of the CS-induced death of SCC-25 cells were related to both aldehydes and redox metals. Consequently, we conducted a study intended, ultimately, to protect the cells against both injurious agents simultaneously. Thus, GSH and DES were added concomitantly prior to the exposure to CS in the presence of saliva and as anticipated an absolute protection against the lethal CS effects was demonstrated, both by rescuing the cells, as noted in the survival assay, and by dropping the carbonyl oxidation level to nearly zero (Figures 8 and 9). The results suggest that the two mechanisms demonstrated act in concert: first, a direct hit of CS-borne aldehydes unrelated to the saliva, then, an attack of aggressive ROS whose production is induced by active metal ions in the saliva which react with CS, causing the synergism. Furthermore, it seems that for some unknown physiological reason, Nature uses saliva to secrete redox metals that in turn may be very injurious to neighbouring biological macromolecules under the ‘wrong circumstances’ unless balanced by antioxidants and/or metal chelators. Two ‘wrong circumstances’ are products of modern life: head and neck radiotherapy and CS. The first results in the destruction of the salivary glands (Nagler *et al*, 1997; Nagler and Baum, 2003) and the other promotes, as we suggest, the induction of OP cancer.

According to recent reports, salivary involvement in the pathogenesis of OP cancer is based on an additional aspect, that is, the inhibitory and destructive effects that CS has on the salivary protective machinery, as it has on enzymes such as LDH, amylase or Acid phosphatase (Table 1

**Table 1:** Inhibitory effects of CS on salivary protective machinery

**Enzyme**	**Initial activity at zero time (U l^−1^)**	**Activity at 3 h (U l^−1^)**	**% of change**	***t*-test**
LDH	340.0±21.1	146.0±23.2	−57.1	*P*<0.004
Amylase	76.2±8.7	50.4±12.7	−33.8	*P*<0.04
ACP	52.0±4.42	12.0±2.5	−77	*P*<0.002
ALP	9.0±1.0	8.6±2.1	−4.5	NS
AST	33.3±6.8	27.6±5.5	−17.2	NS

**Figure 10:**
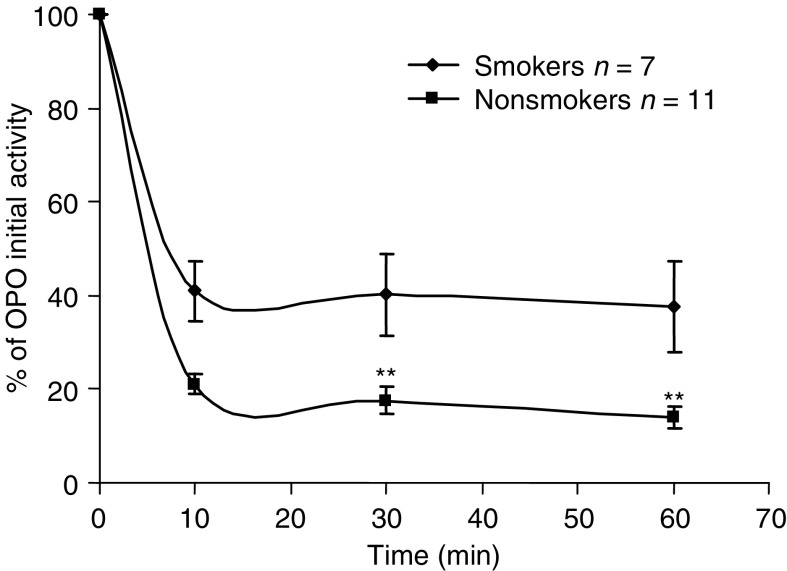
This figure is reprinted with permission from Elsevier published paper: AZ Reznick, I Klein, JP Eiserich, CE (2003) Cross and R Nagler. Inhibition of oral peroxidase activity by cigarette smoke: *in vivo* and *in vitro* studies, FRBS 34(3): 377–384. It delineates the *in vitro* effect of smoking one cigarette on oral peroxidase activity in saliva from seven smokers and 11 nonsmokers. In both groups the enzyme activity was inhibited substantially (by 60–85%) (*P*<0.01).

This table is reprinted with permission from Elsevier published paper: R Nagler, S Lischinsky, E Diamond, N Drigues, I Klein and AZ Reznick (2000) Effect of cigarette smoke on salivary proteins and enzyme activities ABB 379(2): 229–236. As is noted the inhibitory effect of CS on various salivary enzyme activities is differentiated. While it is very substantial on some enzymes as on acid phosphatase (ACP) and lactate dehydrogenase (LDH), it was rather moderate on amylase and nil on alkaline phosphatase (ALP) and on aspartate aminotransferase (AST).

) (Nagler *et al*, 2000, 2001; Reznick *et al*, 2003). Peroxidase is by far the most important antioxidant enzyme in the OP cavity, and as such theoretically serves as the first line of anticarcinogenic defense against free radical-induced OP cancer. It is interesting to note that the possible role of saliva as an anticarcinogenic medium was recently demonstrated in an animal study in which oral cancer was induced by a local carcinogen, 4NQO (Nishioka *et al*, 1981; Dayan *et al*, 1997). Further credence for salivary anticarcinogenic protective capacity was given by Nishioka *et al* (1981) who, using the Ames test, found that saliva inhibited the mutagenicity of another well-known local oral cancer inducer, benzopyrene (Figure 10

).

**Figure 11:**
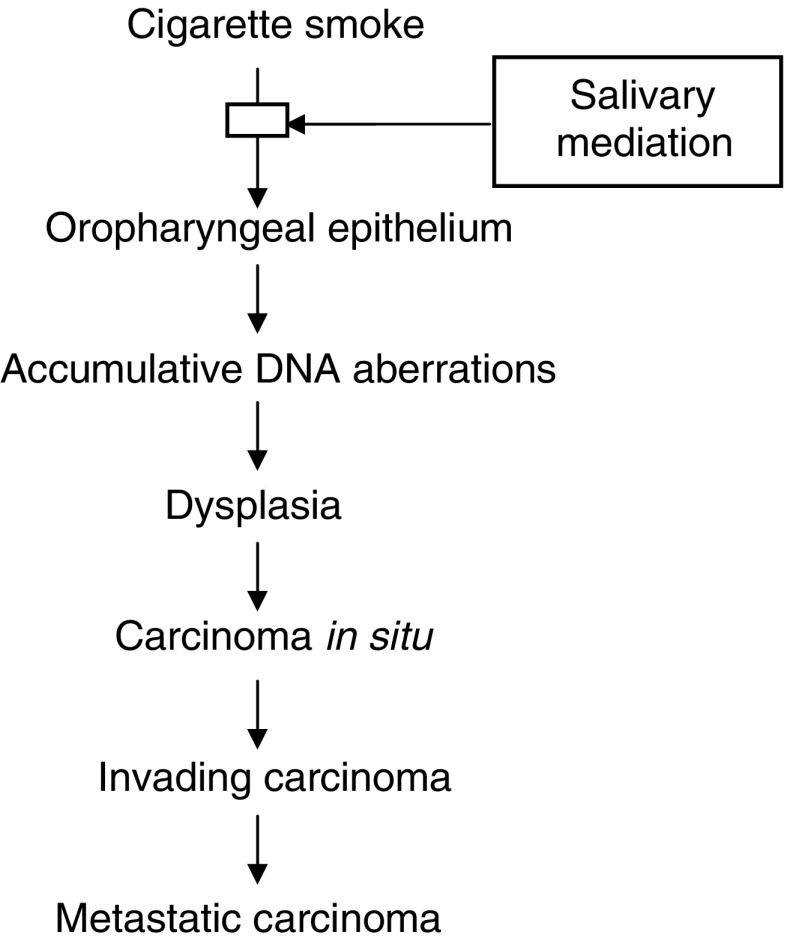
Schematic algorithm in which the suggested step where saliva is involved in the cascade of events which leads from exposure of the OP epithelial cells to CS and ends in the development of a full-blown invasive lethal OP cancer.

In summary, the results presented in the current study delineate the role of saliva in the pathogenesis of OP cancer. A novel lethal synergistic effect of CS and saliva on oral cancer cells is demonstrated. This synergism is based on the reaction between redox active metals in saliva (more so in Sm/Sl saliva) and low reactive free radicals in CS (the salivary related direct pathway). This is a novel concept, recognising that when exposed to CS, salivary behavior is reversed and saliva loses its antioxidant capacity and becomes a potent pro-oxidant milieu. The devastating role of CS-borne aldehydes is demonstrated as well (the non-salivary related direct pathway). Based on the results obtained and on the well-known observation that OP cancer mostly occurs in OP epithelial cells exposed to tobacco and *always* in the presence of saliva, a comprehensive view of the pathogenesis of OP cancer is suggested (Figures 11

and 12

**Figure 12:**
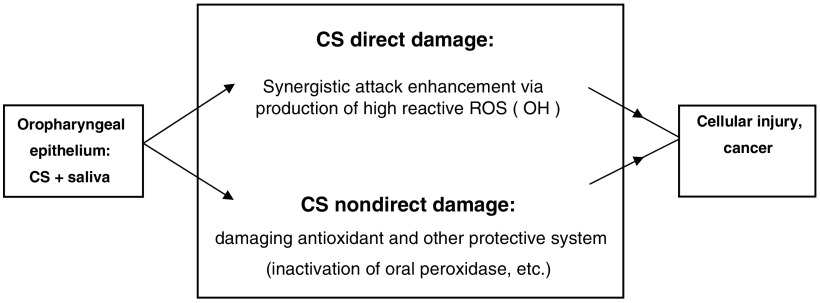
Schematic algorithm delineating the suggested role of saliva as in the pathogenesis of OP cancer, which is inflicted in two pathways. The first is based on a direct damage induced by CS that is mediated by a synergistic injurious effect that it has with saliva, producing highly reactive hydroxyl free radicals. The second is based on a CS nondirect damage that is based on inhibiting salivary anticarcinogenic capacity, such as that inflicted by inhibition of the activity of salivary peroxidase (the most important salivary antioxidant enzyme).

). It is also based on recent studies which demonstrate an inhibition induced by CS of various salivary components. Of those components, the most important are those of the salivary protective machinery, such as peroxidase, which is the most important salivary antioxidant enzyme (the salivary related indirect pathway). When the activity of peroxidase is inhibited, H_2_O_2_ is not removed and its level is substantially increased. That adds credence to the currently suggested metal-related OP cancer pathogenesis and is in full accord with the conclusions published recently by Kawanishi *et al.* (2002), which suggest a mechanism for carcinogenesis induced by various metals, such as iron and copper. Accordingly, various metallic compounds are capable of causing oxidative DNA damage in the presence of H_2_O_2_, and produce highly reactive species such as hydroxyl-free radicals that in turn result in oxidative DNA damage. Furthermore, DNA repair systems are also sensitive targets for carcinogenic metals. In addition, in a very important recent article, Kasprzak clearly declared that the two essential metals, iron and copper, are the strongest carcinogens and that they mediate their carcinogenesis through oxidative damage to the DNA. He stated, ‘…owing to its relative abundance and high capacity to activate oxygen, iron displaced from natural sources, eg, by another metal or toxic insult, is often considered as the ultimate carcinogen…’ (Kasprzak, 2002). Moreover, the role of metals in this carcinogenesis is occasionally mediated by inhibition of DNA repair. Finally, it is also mediated by the metal-induced alteration of the proper progression of the cell cycle and/or apoptosis. That is because ROS serve as physiological signal transduction messengers in controlling gene expression, including oncogenes and tumour suppressor genes, and as previous noted, metals substantially change the balance of ROS produced (Kasprzak, 2002). Taken together, all the above may point to the possible pivotal role of saliva in the pathogenesis of OP cancer. Moreover, it is well known that there is a substantial higher rate of detecting new OP malignant lesions following the treatment of previously primary lesions, which most often includes head and neck radiotherapy and a subsequent xerostomia. However, under these circumstances, the xerostomia may contribute to cancer development (paradoxically as it seems) since salivary antioxidants are thus reduced in the oral cavity. In any case, in many cases, following the first diagnosis of OP, patients stop smoking and as previously explained saliva, if not exposed to CS, does not present its promalignant but rather its antimalignant nature. We believe that the novel concept presented in the current study may open avenues for developing new means for prevention of OP cancer.
